# Hemodynamics and cardiac autonomic modulation after an acute concurrent exercise circuit in older individuals with pre- to established hypertension

**DOI:** 10.6061/clinics/2021/e1971

**Published:** 2021-01-11

**Authors:** Ricardo Cordeiro, Pedro Augusto Mira, Walace Monteiro, Felipe Cunha, Mateus C. Laterza, Linda S. Pescatello, Daniel G. Martinez, Paulo Farinatti

**Affiliations:** I Programa de Graduacao em Ciencias da Atividade Fisica, Universidade Salgado de Oliveira, Niteroi, RJ, BR; II Laboratorio de Atividade Fisica e Promocao da Saude (LABSAU), Instituto de Educacao Fisica e Desportos, Universidade do Estado do Rio de Janeiro, Rio de Janeiro, RJ, BR; III Departamento de Fisiologia e Farmacologia, Laboratorio de Ciencia do Exercicio, Universidade Federal Fluminense, Niteroi, RJ, BR; IV Unidade de Investigacao Cardiovascular e Fisiologia do Exercicio, Hospital Universitario e Faculdade de Educacao Fisica e Desportos, Universidade Federal de Juiz de Fora, Juiz de Fora, MG, BR; V Department of Kinesiology, University of Connecticut, CT, USA

**Keywords:** Blood Pressure, Cardiac Output, Systemic Vascular Resistance, Autonomic Nervous System, Elderly

## Abstract

**OBJECTIVES:**

Few studies have investigated whether post-exercise hypotension (PEH) after concurrent exercise (CEX) is related to changes in cardiac output (Q) and systemic vascular resistance (SVR) in older individuals. We tested whether PEH after a single bout of CEX circuits performed in open-access facilities at the Third Age Academies (TAA) in Rio de Janeiro City (Brazil) would be concomitant with decreased Q and SVR in individuals aged ≥60 years with prehypertension. Moreover, we assessed autonomic modulation as a potential mechanism underlying PEH.

**METHODS:**

Fourteen individuals (age, 65.8±0.9 y; systolic/diastolic blood pressure [SBP/DBP], 132.4±12.1/72.8±10.8 mmHg; with half of the patients taking antihypertensive medications) had their blood pressure (BP), heart rate (HR), Q, SVR, HR variability (HRV), and spontaneous baroreflex sensitivity (BRS) recorded before and 50 min after CEX (40-min circuit, including seven stations of alternate aerobic/resistance exercises at 60-70% HR reserve) and non-exercise control (CONT) sessions. The study protocol was registered in a World Health Organization-accredited office (Trial registration RBR-7BWVPJ).

**RESULTS:**

SBP (Δ=−14.2±13.1 mmHg, *p*=0.0001), DBP (Δ=−5.2±8.2 mmHg, *p*= 0.04), Q (Δ=−2.2±1.5 L/min, *p*=0.0001), and BRS (Δ=−3.5±2.6 ms/mmHg; *p*=0.05) decreased after CEX as compared with the CONT session. By contrast, the HR increased (Δ=9.4±7.2 bpm, *p*<0.0001), and SVR remained stable throughout the postexercise period as compared with the CONT session (Δ=0.10±0.22 AU, *p*=0.14). We found no significant difference between the CEX and CONT with respect to the HRV indexes reflecting autonomic modulation.

**CONCLUSION:**

CEX induced PEH in the older individuals with prehypertension status. At least in the first 50 min, PEH occurred parallel to the decreased Q and increased HR, while SVR was not different. The changes in autonomic outflow appeared to be unrelated to the acute cardiac and hemodynamic responses.

## INTRODUCTION

Hypertension is a major risk factor for cardiovascular diseases, accounting for approximately 8 million deaths each year (1). The prevalence of hypertension among the elderly is a major public health issue, given the increasing proportion of the elderly in the world population (2). Therefore, strategies to improve the management of hypertension in the elderly are justified. Regarding non-pharmacological strategies, evidence shows that physical exercise contributes to lower blood pressure (BP), particularly in individuals with prehypertension status to established hypertension (3,4).

A single bout of exercise has been shown to reduce BP for up to 24 h as compared with control values (4-10). This phenomenon is referred to as post-exercise hypotension (PEH), and its importance has been acknowledged (6,). First, prolonged PEH during daytime may be relevant for patients with prehypertension status or hypertension, as this is usually the period when they exhibit the highest BP values (11,12). Besides this immediate benefit, some authors have suggested that PEH could be a potential determinant of long-term BP reductions that result from exercise training (13,14). A better understanding of the mechanisms underlying PEH would be important to optimize the antihypertensive effects of exercise.

PEH has been observed after different exercise modalities and in several populations, from young adults to the elderly, sedentary or physically trained, and with normal or high BP (3,6,15,16). In addition, strong evidence indicates that isolated aerobic (4,17) and resistance exercise (18) reduce BP chronically. However, from a practical perspective, exercise sessions designed to lower BP and promote general health (19) rarely include exclusive aerobic or resistance exercise. In fact, these modalities are often performed within the same exercise session (*e.g.*, combined or concurrent exercise). Emerging evidence shows that chronic concurrent exercise also lowers BP (20,21), and at least one trial observed a moderate correlation between acute and chronic decreases in systolic BP (SBP) after the training modality (22). However, in comparison with studies on isolated aerobic and resistance exercises, studies that addressed whether PEH occurs after concurrent exercise are scarce (23-25), particularly in older adults (22,26-28).

In the city of Rio de Janeiro, a local policy named “Third-Age Academies” (TAA) was implemented to provide supervised physical activities to older people at open-access facilities such as squares and parks (29). In 2015, >230 units with 300-400 formally registered patients were established, including approximately 40,000 elderly attendees. The training equipment was specifically designed for older individuals and routines that consisted of concurrent training using body mass or fixed loads to increase intensity. Promising results from a preliminary trial from our group showed that acute concurrent exercise in TAAs induced PEH in the elderly with prehypertension status to established hypertension (27). However, the mechanisms underlying this acute response were not addressed in that study.

Central and peripheral components are involved in PEH, including lower sympathetic activity and sustained peripheral vasodilation (5,11). These factors produce changes in cardiac output (Q) and systemic vascular resistance (SVR) during post-exercise recovery (5,6). However, their relative contributions to PEH appears to depend on the type of population and exercise characteristics (8,11,15,21,23). Some evidence indicates that decreased Q values due to a reduction in stroke volume (SV) is a major mechanism underlying PEH in the elderly, but this response has been reported only in trials applying aerobic (8,15) or resistance exercises (30) performed in isolation. To date, the relative contributions of Q and SVR to PEH after acute concurrent exercise in older persons with prehypertension status have not been investigated.

In addition, both a reduction (5,11,31) and an increase in sympathetic activity with or without reduction in parasympathetic activity (23,32,33) concomitant with PEH have been reported. Previous studies showed that a combination of central and peripheral factors occurs concomitantly with hemodynamic responses post-exercise, particularly reduced sympathetic nerve activity and local vasodilation (5,9). Some evidence indicates that baroreflex resetting and reduced efferent cardiac sympathetic activity may play a role in reducing both SV and vasoconstriction post-exercise (7,9,11). Briefly, changes in Q and SVR during PEH might be, in some extent, influenced by fluctuations in autonomic control. However, few trials assessed BP along with hemodynamic and autonomic markers after acute concurrent exercise, including individuals with normal BP (23,34). The only trial that included older patients with hypertension failed to show the associations between PEH and autonomic changes (26), and further studies are necessary to confirm these findings.

Given these knowledge gaps, the present study tested the hypothesis that Q would be reduced after a single bout of concurrent exercise circuit applied in TAAs, which would be useful for explaining the occurrence of PEH in individuals aged >60 years with prehypertension status. In addition, postexercise cardiac autonomic modulation was assessed to ascertain whether lowered BP and Q would be centrally mediated by changes in sympathetic or parasympathetic outflows.

## METHODS

### Ethical considerations

All the participants signed an informed consent form before enrollment in the study, which was approved by the local institutional research committee (Process CAAE 30184114.4.0000.5289). Moreover, as part of a broader clinical trial, the study protocol was registered in a World Health Organization-accredited office (Trial registration RBR-7BWVPJ).

### Participants

The study included 14 older individuals (12 women) who did not practice regular physical activity (<2 days/week) within 2 months prior to enrollment. Participants were recruited by advertisements in TAAs, which consisted of open access exercise public facilities in the city of Rio de Janeiro, where supervised concurrent exercise circuits are dispensed to senior citizens. The recruitment took place at the time when new participants were enrolled in the TAAs so that they had no prior familiarity with the exercise circuit.

The participants were aged ≥60 years, had a prehypertension status (35) (systolic BP >120 mmHg or diastolic BP >80 mmHg), and were cleared by their physicians to practice physical activities. All the participants were dippers according to the Brazilian Society of Cardiology criteria (36). The exclusion criteria were smoking, cardiac or chronic kidney disease, stroke within the last year, orthopedic problems compromising exercise performance, diabetes, and use of medications influencing autonomic modulation activity and systemic vascular resistance (*e.g.*, alpha-agonists, alpha-blockers, beta-blockers, third-generation beta-blockers, and calcium channel blockers). Medications that did not influence HR or autonomic control were accepted, such as diuretics and AT1 receptor angiotensin and angiotensin-converting-enzyme inhibitors.

### Experimental Design

This trial was comprised of four visits on separate days. On the first visit, the volunteers underwent clinical screening to determine their eligibility for the study, including 24-h ambulatory BP monitoring (ABPM). On the second visit, body mass and height were assessed, and the participants were familiarized with the exercises performed during the circuit sessions at the TAAs. On the third and fourth visits, they performed either control (CONT) or concurrent exercise (CEX) sessions interspersed with 48-72h, in a randomized counterbalanced order. BP (oscillometric method) and RR intervals (RRi) were recorded 15 min before and throughout the 50 min after the CONT and CEX sessions. All the procedures were performed in a laboratory temperature-controlled room (−22°-25°C) and at the same time of the day (3:00 to 4:00 PM) to minimize the circadian effects on BP and cardiac autonomic modulation.

### Concurrent Exercise and Control Sessions

The standard circuit applied in CEX included the following aerobic and resistance exercises, as detailed elsewhere (27): 1) warm-up and stretching (5-10 min); 2) calisthenics (5-10 min) to increase heart rate (HR) up to 50% of the age-predicted heart rate reserve (HRR); 3) the main phase, consisting of a single circuit alternating seven stations with aerobic and resistance exercises (around 40 min) performed at moderate intensity (60-70% HRR). The seven stations were as follows: first station (aerobic, around 5 min)-walk simulation; second station (resistance, 1 set of 15 repetitions, around 6 min)-leg extension, seated bench press, squat, lat pull-down, seated leg curl, and seated shoulder press; third station (aerobic, around 5 min)-ski simulation; fourth station (resistance, 1 set of 15 repetitions, around 6 min)-leg extension, seated bench press, squat, lat pull-down, seated leg curl, and seated shoulder press; fifth station (aerobic, around 5 min)-walk simulation; sixth station (resistance, 2 sets of 15 repetitions, around 6 min)-seated leg press, seated rowing, and plantar flexion; and seventh station (aerobic, around 5 min)-ski simulation. All the exercises were performed either at articulated machines or freely, using body mass as loads.

At the end of each circuit station, the rating of perceived exertion (RPE) was assessed using the Borg CR-10 scale. HR and RRi were continuously assessed using an HR monitor (RS800cx, Polar). The duration of exercise and average HRR were recorded, and the training impulse (TRIMP) in the CEX sessions was calculated to ensure that exercise intensity and volume were similar across the participants (27,37). TRIMP integrates in a single variable both intensity and volume of training, determined by multiplying the duration of exercise bout (TD) by the mean HRR, as follows: (1) TRIMP, a.u. (men)=TD×%HRR×0.64×*e* ^(1.92x%HRR)^ and (2) TRIMP, a.u. (women)=TD×%HRR×0.86×*e* ^(1.67x%HRR)^ (37). In the non-exercise CONT, the participants remained seated at rest, talking, or reading.

### Pre-participation health screening and anthropometric assessment

A physician screened all the participants for health status, including personal data, clinical status and medical history, current medication, and habitual physical activity within the last 2 months. Body mass was measured to the nearest 1g by using a digital scale (Welmy, SP, Brazil), and height was measured in centimeters by using a wall-mounted stadiometer (AMB, SP, Brazil).

### Measurements

#### BP and hemodynamic outcomes

As a criterion of eligibility, BP was measured for 24h (Spacelabs Medical, Redmond, WA, USA), and a certified cardiologist determined whether the participants exhibited prehypertension to established hypertension. A trained researcher attached the ABPM device to the patient at the end of the first visit to the laboratory. Measurements were taken every 20 min during the daytime (between 5:00 AM to 10:00 AM) and every 30 min during sleep (between 10:00 PM to 7:00 AM). In the first hour, an automated Omron 705IT device (Omron Healthcare, Kyoto, Japan) assessed BP in the contralateral arm to validate the ABPM measurements.

Prior to the CONT and CEX, a trained researcher measured BP using the same automated Omron 705IT device (Omron Healthcare, Kyoto, Japan) placed around the right arm. The participants remained at rest for 15 min in the supine position to allow the stabilization of cardiovascular variables in a temperature-controlled laboratory room (−22°C to 25°C). Thereafter, BP was measured three times (1-min intervals) and averaged. The differences between the BP measurements obtained at rest using the oscillometric device did not exceed 5% of the mean ABPM during daytime. After both experimental sessions, BP was measured for 50 min (at 20, 30, 40, and 50 min) after the same procedures were applied in the pre-exercise assessment.

The changes in cardiac outcomes (Q and SV) and SVR were estimated through beat-by-beat photoplethysmography (Finometer Pro, Finapres Medical Systems, Arnhem, The Netherlands) in the supine position, over 15 min at rest and 50 min after CONT and CEX in a temperature-controlled laboratory room (−22°C to 25°C). The finger cuff was positioned at the middle finger of the left hand. SV was derived from pressure signals, and Q was determined as the product between SV and HR. SVR was calculated using the model flow method, which is an automated method that computes aortic blood flow from the arterial pressure wave by simulating a nonlinear, time-varying three-element model of aortic input impedance. SVR was incorporated into the calculation data related to sex, age, height, and body mass using the BeatScope 1.0 software (Finapres Medical Systems).

#### Cardiovascular autonomic modulation

Concomitantly to the beat-by-beat BP monitoring, HR and HR variability (HRV) were recorded using a telemetric HR monitor (RS800cx, Polar). The RRi data were downloaded onto a PC by using the Polar Precision Performance software (Polar) and analyzed using the Kubios HRV software (Biomedical Signal Analysis Group, Kuopio, Finland), considering the last 5 min of the 15-min rest and 10-min window along post-exercise recovery. The sampling frequency was 1000 Hz, and signal artifacts were filtered by excluding RRi with differences of >20% from the preceding RRi (38). Filtering was performed in <1% of the sequences for each subject.

The time-domain analysis performed consisted of measures of RRi (average of all normal RRi) and rMSSD (square root of the sum of the successive differences between adjacent normal R-R intervals squared). In the frequency domain, spectral analysis time series were processed by fast Fourier transform using Welch’s method and a Hanning window with a 50% overlap. The beat-by-beat RRi series were then converted into equally spaced time series with 512-ms intervals using cubic spline interpolation (39). The power spectrum density function was integrated in the two classical frequency bands as follows: 1) low frequency band (LF: 0.04-0.15 Hz) and 2) high-frequency band (HF: 0.15-0.40 Hz) (39). HF was used as an index of vagal modulation, whereas LF was considered representative of both sympathetic and parasympathetic nervous system influences (39,40). The spectral values were expressed as normalized units (n.u.). The LF/HF ratio was adopted as a marker of sympathovagal balance.

The SBP and pulse interval data derived with finger photoplethysmography (Finometer, FMS, Amsterdam, The Netherlands) were exported into the Heart Scope v 1.3.0.1 software (AMPS, New York, NY). The baroreflex sensitivity (BRS) was analyzed using the alpha index from the low-frequency band (α-LF) of the beat-by-beat SBP and pulse interval (41). Only the detected spectral gains with a coherence > 0.5 (arbitrary threshold) were accepted.

### Statistical analyses

The statistical power estimated using *post hoc* analysis was 0.79 (G*Power 3.1.5, Universitat Dusseldorf, Dusseldorf, Germany; effect size *f*: 0.30; *α* error probability: 0.05; number of groups: 2; correlation among repeated measures: 0.5; nonsphericity correction: 1; number of measurements: 5). Data normality was checked using Kolmogorov-Smirnov tests and logarithmic transformations performed whenever necessary. The results are expressed as mean±standard deviation (SD). Student *t* tests for paired samples were used to compare environmental (temperature and air humidity) and hemodynamic and autonomic markers at rest between CONT and CEX. Within-between differences across the experimental conditions regarding hemodynamic and autonomic outcomes were tested with two-way analysis of variance with repeated measures, followed by Sidak *post hoc* tests in the event of significant *F* ratios. Statistical calculations were performed using SPSS 20.0 (SPSS Inc., Chicago, IL, USA), and the probability level was fixed at *p*≤0.05.

## RESULTS

Table 1 depicts the demographic, physical, and hemodynamic characteristics and medications of the subjects enrolled in the study. The participants were overweight, either with BP compatible with prehypertension or medication. Table 2 shows the BPs, HRV indexes, and BRS measured before CONT and CEX, with no significant differences detected between the conditions.

As total exercise duration and environmental conditions might influence BP, these variables were compared between the experimental conditions. No significant differences in session duration were found between CONT and CEX (40±1 *vs*. 41±1 min; *p*=0.77), environment temperature (25°C±1°C *vs*. 25°C±1°C; *p*=0.92), or relative humidity (57%±1% *vs*. 57%±2%; *p*=0.97). The exercise volume elicited by the CEX as reflected by TRIMP was similar between the participants, with a small coefficient of variation (25.4±0.5 a.u. or CV=2%), and RPE assessed with the Borg CR-10 scale (4.2±0.2 or CV=5%).

**Figure 1:**
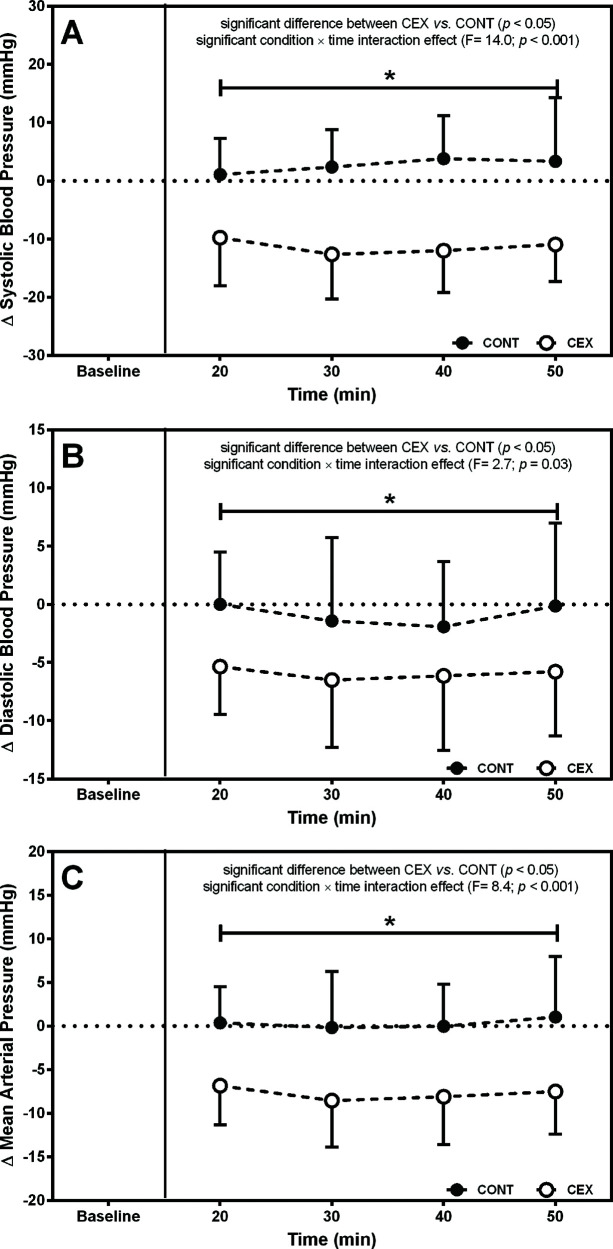
Changes (deltas) in systolic blood pressure (SBP), diastolic blood pressure (DBP), mean arterial pressure (MAP) throughout the 60-min recovery after concurrent exercise (CEX) and non-exercise control (CONT) sessions (n=14; mean±standard deviation). *p<0.05 *versus* the CONT.

Figures 1 and 2 depict the changes in BP and hemodynamic data after the two experimental conditions, respectively. On average, SBP (Δ=−14.4±13.1 mmHg; *F*=14.00, *p*=0.0001), DBP (Δ=−5.1±8.2 mmHg; *F*=2.70, *p*=0.03), and mean arterial pressure (MAP) (Δ=−8.0±8.7 mmHg; *F*=8.74, *p*=0.0001) were lower after CEX than after CONT. Concomitantly, significant reductions in Q (Δ=−2.2±1.5 L/min; *F*=20.20, *p*<0.0001) and SV (Δ=−45.3±28.0 mL; *F*=26.23, *p*<0.0001) but increased HR (Δ=9.4±7.2 bpm; *F*=13.09, *p*<0.0001) were found in CEX as compared with CONT. No significant difference between conditions was detected for SVR during the post-exercise recovery (Δ=0.10±0.22 a.u.; *F*=0.03, *p*=0.14).

Finally, Table 3 presents the variations of the HRV indexes and BRS during post-exercise recovery in the CONT and CEX groups. Compared with CONT, CEX elicited reductions in RRi and BRS of −123±128.2 ms (*F*=14.7; *p*=0.001), and −3.5±2.6 ms/mmHg (*F*=4.1; *p*=0.05), respectively. A significant condition × time interaction showed that the differences between CONT and CEX decreased over time for RRi (*F*=10.0; *p*=0.001) and BRS (*F*=7.0; *p*=0.014). By contrast, no significant differences were found between the conditions for rMSSD (Δ=−9±15.3 ms; *F*=1.85, *p*=0.12), LF (Δ=2.2±20.5 n.u.; *F*=1.09, *p*=0.36), HF (Δ=−2.2±20.8 n.u.; *F*=1.17, *p*=0.32), or LF/HF (Δ=0.5±2.2; *F*=1.82, *p*=0.13).

## DISCUSSION

This study measured BP and hemodynamic and autonomic outcomes for 50 min after CEX performed in TAAs and non-exercise control sessions in older individuals with prehypertension status. The main findings were as follows: a) CEX induced PEH, whereas CONT did not, and b) PEH was mainly due to a reduction in Q as a consequence of a decrease in SV and increase in HR. Reduced Q was concomitant with attenuated BRS, which is suggestive of lowered vagal modulation after CEX, albeit without significant changes in the HRV indexes.

These findings support the hypothesis that PEH after CEX occurs in older individuals with prehypertension to established hypertension at rest, and that the occurrence of PEH is paralleled by decreases in Q and SV. The occurrence of PEH in older individuals has been previously reported, which is in agreement with our findings (8,22,26,27,42). In addition, some previous trials with this population indicated that reductions in Q and SV are not completely compensated by an increase in SVR (8,15). The degree of hypertension is acknowledged to influence the magnitude of PEH. Patients with higher BP at rest are more likely to experience greater reductions after exercise (6,10,11). In addition, the degree of hypertension may also influence the potential mechanisms explaining PEH. A prior study suggested that increases in Q in individuals with normal BP and decreases in Q in those with high BP might occur in PEH (7). In addition, the mechanisms underlying PEH after acute exercise may differ between young and older individuals; a decrease in Q seems to be more frequent in the elderly (15). In short, in older individuals with high BP, PEH appears to be due to a reduction in Q. However, previous studies investigated aerobic or resistance exercises in isolation. The present study adds to the current knowledge by indicating that similar mechanisms may be at the origin of PEH after acute CEX in older individuals with prehypertension status.

Several mechanisms may explain the occurrence of PEH, including lower sympathetic activity and sustained peripheral vasodilation (5,11). These effects may induce isolated or concomitant reductions in myocardial contractility and venous return (11,43). The present study did not detect alterations in HRV indexes after CEX and CONT, which is in disagreement with a prior experiment that assessed BP after acute concurrent exercise in young participants (23). On the other hand, the attenuated BRS is suggestive of lower vagal activation after CEX, which might be a physiological response to offset the reductions in BP and to counteract the baroreflex resetting post-exercise (5,7). The fact that the HR remained elevated during the entire recovery period after CEX reinforces this premise. The decrease in HR during post-exercise recovery depends on the removal of metabolites and reduction in body temperature, both of which are related to attenuated parasympathetic reactivation and sympathetic withdrawal (44,45). Augmented HR after exercise has been attributed to a persistent increase in sympathetic outflow and decrease in parasympathetic outflow (23,30,43). Collectively, these findings suggest that a slight sympathovagal increase may have occurred in parallel with the reductions in BP, although changes in LF and LF/HF ratio were not detected.

Moreover, decreases in Q and SV have long been reported to be related to systolic dysfunction (6). However, Rondon et al. (8) showed that a reduction in left end-diastolic volume explained the decreases in SV and Q after aerobic exercise in older individuals with hypertension. In addition, no change in ejection fraction or fractional shortening occurred post-exercise. Taken together, these results suggest that the reduction in SV was not due to an impairment in systolic function following exercise. Further research is needed to clarify this specific hemodynamic response in older patients with prehypertension and to better ascertain the potential contribution of changes in autonomic modulation to produce PEH.

Previous studies showed a reduction in SVR after exercise results from a blunted neuronal firing of cardiovascular sympathetic neurons due to resetting in the operating point of the arterial baroreflex (5,6,11). Indeed, a decrease in the slope of the linear regression between muscle sympathetic nerve activity and calf vascular resistance after a bout of aerobic exercise was reported (31), indicating lower neurovascular transduction. However, while sustained vasodilation with lower SVR has been reported after acute exercise in young and middle-aged individuals (5,11,15,23), this finding was not confirmed by trials that applied isolated aerobic bouts in older participants (8,15,46). Our data reinforced this premise, suggesting that the PEH that occurred after the present circuit of CEX was not due to a reduction in SVR. We can speculate that hypertension and aging would lead to vascular stiffening and remodeling, predisposing patients to endothelial dysfunction (8,15,47). These vascular impairments may restrain the post-exercise vasodilation capacity in seniors with prehypertension to established hypertension, which may explain why the SVR remained unaltered, with a greater dependence of the PEH on changes in Q and SV (5,8,11,15).

In terms of practical implications for public health, the decreases in SBP and DBP after CEX were clinically relevant, being approximately 14 and 5 mmHg, respectively. In a previous trial, we demonstrated that CEX usually performed in TAAs was capable of eliciting PEH in older individuals for several hours, especially when the resting SBP was high (27). Given the potential impact of the PEH magnitude on chronic BP reductions (4,11), we can speculate that such an acute hypotensive effect may contribute to the long-term decrease in BP. Furthermore, reductions in this order seem to diminish the risk of cardiovascular mortality and morbidity (3,4). Hence, the CEX circuit performed at TAAs emerges as a low-cost, effective intervention aimed at improving BP control among senior citizens. Therefore, future studies should investigate the hypothesis that CEX training performed at TAAs would reduce the BPs of the attended seniors, particularly those with prehypertension status to established hypertension at rest.

This study has a major limitation. The noninvasive measurements of SV and Q by means of photoplethysmography (*e.g.*, Finometer) have been criticized for its potential underestimation in comparison with values obtained through other techniques such as CO_2_ rebreathing or Doppler ultrasonography (48,49). However, studies have certified the reliability and validity of the model flow method using Finometer to assess changes (or deltas) in SV and Q at resting conditions in research settings and populations, including the elderly (50,51). Moreover, SVR has been estimated based on these variables and not directly measured. The possibility of type II error due to the high inter-individual variability of data within a relatively small sample must be acknowledged, particularly with regard to HRV outcomes. Finally, the participants in the present study were primarily women; the application of our data to older men should be made with caution.

In conclusion, in older individuals with prehypertension, CEX performed in the TAAs induced PEH as compared with the non-exercise control sessions. At least in the first 50 min of post-exercise recovery, the acute decrease in BP was concomitant with the reductions in Q and SV, while SVR did not change. The role of the change in autonomic outflow as a potential mechanism underlying these responses is uncertain. However, in the present study, the after-effects of exercise on Q and SV were concomitant with the reduction in BRS and increased HR, which are suggestive of vagal withdrawal and increased sympathetic outflow, even though no changes in autonomic modulation reflected by the HRV indexes were found. Further studies are warranted to confirm these findings and to investigate whether the CEX circuit routines applied in the TAAs might contribute to the chronic reduction in BP and/or autonomic adaptations in older participants with prehypertension status.

## AUTHOR CONTRIBUTIONS

Cordeiro R was responsible for the investigation (data collection), formal analysis (statistics), and manuscript drafting, writing, and review. Mira PA was responsible for drafting, writing, and reviewing the manuscript. Monteiro W was responsible for the conceptualization, methodology, investigation (data collection), and manuscript drafting, writing, and review. Cunha F was responsible for the formal analysis (data synthesis) and manuscript drafting, writing, and review. Laterza MC was responsible for conceptualization, methodology (study design), and manuscript drafting, writing, and review. Pescatello LS was responsible for the manuscript drafting, writing, editing, and review. Martinez DG was responsible for the methodology (study design), and manuscript drafting, writing, and review. Farinatti P was responsible for the conceptualization, data curation, formal analysis (synthesize data), funding acquisition, methodology (study design), project administration, resources, supervision, and manuscript drafting, writing, editing, and review.

## Figures and Tables

**Figure:**
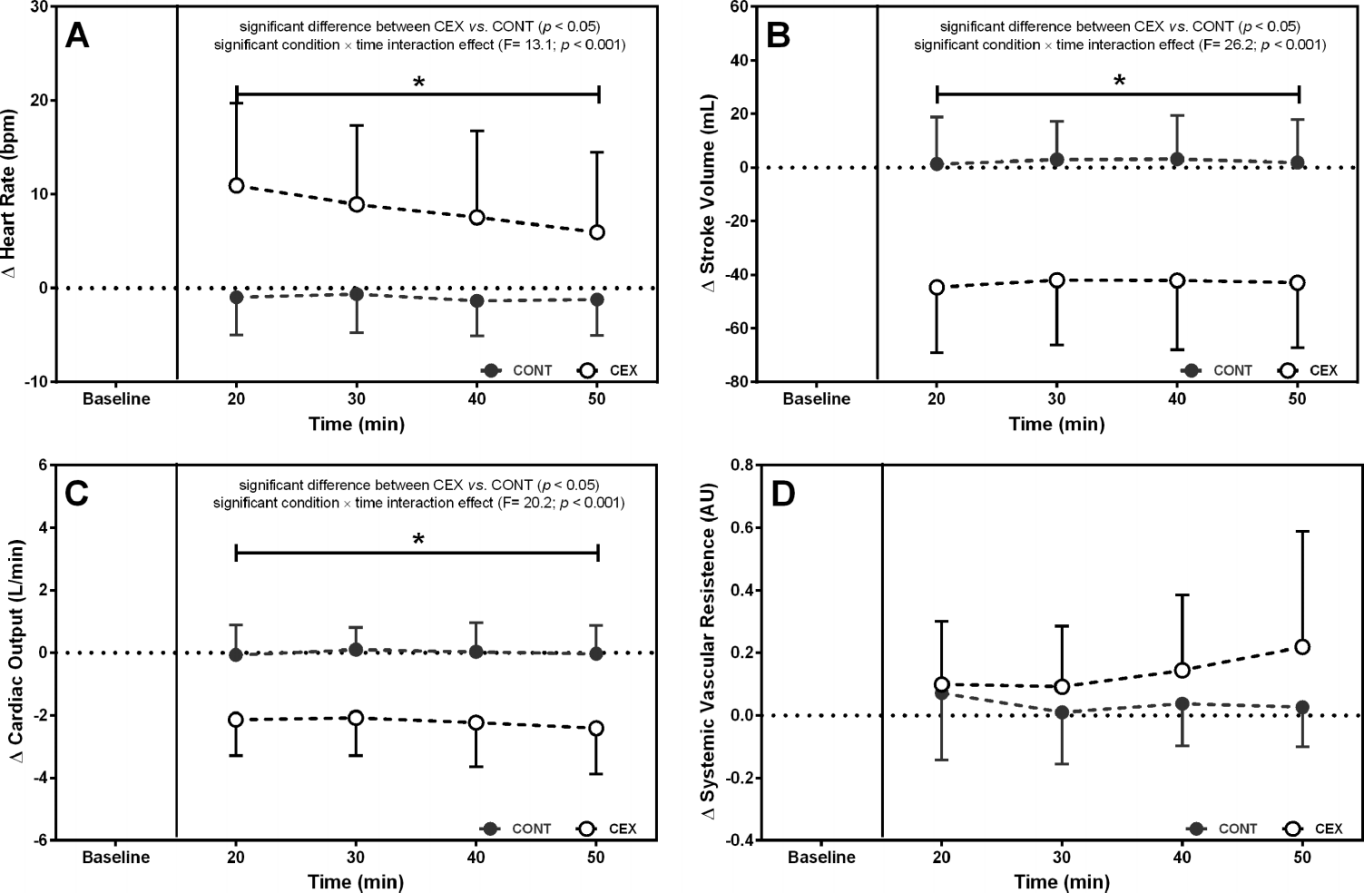
Changes (deltas) in cardiac output (A), systemic vascular resistance (B), stroke volume (C), and heart rate throughout the 60-min recovery (D) after the concurrent exercise (CEX) and non-exercise control (CONT) sessions (n=14; mean±standard deviation). *p<0.05 compared with the CONT.

**Table 1:** Demographic, physical, and hemodynamic characteristics and medications of the individuals enrolled in the study (n=14).

Variable	Mean±SD
Female sex, n (%)	12 (85.7)
Age (y)	65.8±4.9
Body mass (kg)	76.8±11.2
Height (cm)	164.1±6.5
BMI (kg/m^2^)	30.5±7.6
SBP oscillometric (mmHg)	132.4±12.0
DBP oscillometric (mmHg)	72.8±10.1
MAP oscillometric (mmHg)	92.5±10.5
HR (bpm)	73.9±9.7
24-h SBP (mmHg)	126.7±6.4
24-h DBP (mmHg)	68.8±4.6
24-h MAP (mmHg)	88.4±2.7
Medication	
Diuretic+AT1 receptor angiotensin II blocker or ACE inhibitor	2
AT1 receptor angiotensin II blocker	3
ACE inhibitor	2
Without drug therapy	7

ACE, angiotensin-converting enzyme; BMI, body mass index; SBP, systolic blood pressure; DBP, diastolic blood pressure; MAP, mean arterial pressure; HR, heart rate; AT1, receptor angiotensin.

**Table 2:** Blood pressure and cardiac autonomic modulation at rest before the control (CONT) and circuit exercise (CEX) sessions (n=14).

Variable	CONT	CEX	*p-*value
SBP (mmHg)	126.5±7.9	126.3±8.7	0.77
DBP (mmHg)	71.8±8.3	70.6±4.8	0.32
MAP (mmHg)	90.0±7.5	89.1±5.1	0.30
HR (bpm)	67.7±4.2	69.2±5.9	0.38
R-R interval (ms)	898.0±61.9	876.8±121.7	0.51
rMSSD (ms)	22.9±14.7	21.6±13.5	0.77
LF (n.u.)	60.4±16.6	66.1±12.4	0.11
HF (n.u.)	39.6±16.6	33.9±12.1	0.11
LF:HF	2.2±2.2	2.4±1.3	0.66
BRS (ms/mmHg)	9.2±5.4	8.9±3.3	0.87

Values are expressed as mean±SD. SBP: systolic blood pressure; DBP: diastolic blood pressure; MAP: mean arterial pressure; HR: heart rate; rMSSD: square root of the sum of successive differences between the adjacent normal R-R interval squared; HF: high-frequency power band (0.15-0.40 Hz); LF: low-frequency power band (0.04-0.15 Hz); LF/HF ratio reflecting sympathovagal balance; BRS: spontaneous baroreflex sensitivity.

**Table 3:** Changes in the heart rate variability indexes and spontaneous baroreflex sensitivity after control (CONT) and concurrent exercise (CEX) sessions (n=14).

	Postexercise recovery (min)			
Variable	20	30	40	50
Δ RRi (ms)				
CONT	2.1±30.9	2.3±37.5	6.9±26.6	7.3±34.7
CEX	−144.3±127.4*	−120.7±126.9*	−111.2±141.1*	−97.3±147.7*
Δ rMSSD (ms)				
CONT	1.2±8.8	6.1±17.5	2.2±8.9	0.8±4.0
CEX	−7.6±15.4	−6.0±15.1	−5.6±15.7	−6.4±12.7
Δ LF (n.u.)				
CONT	0.4±16.2	1.2±13.2	1.8±13.7	3.9±13.5
CEX	8.6±16.5	2.6±18.3	3.3±18.3	1.7±16.1
Δ HF (n.u.)				
CONT	−0.4±16.2	−1.2±13.2	−1.8±13.7	−3.9±13.5
CEX	−8.6±16.1	−2.6±18.5	−3.3±15.9	−1.7±17.0
Δ LF/HF ratio				
CONT	0.1±1.6	0.1±1.3	0.1±1.2	0.5±1.3
CEX	1.5±1.8	0.6±2.1	0.5±1.9	0.4±2.2
Δ BRS (ms/mmHg)				
CONT	−0.8±2.4	−0.9±4.1	−0.6±4.0	−0.3±3.5
CEX	−4.9±2.8*	−4.7±2.6*	−4.3±2.4*	−4.1±2.5*

Values are expressed as mean±SD. Delta (*Δ*): average change between the post- and pre-intervention values. RRi: average of all normal RR intervals; rMSSD: square root of the sum of the successive differences between the adjacent normal R-R intervals squared; HF: high-frequency power band (0.15-0.40 Hz); LF: low-frequency power band (0.04-0.15 Hz); LF/HF ratio reflecting sympathovagal balance; BRS: spontaneous baroreflex sensitivity.

* Significantly different compared with the CONT (*p*<0.05).
